# Leukocytosis and C-Reactive Protein May Predict Development of Secondary Cerebral Vasospasm in Patients with Aneurysmal Subarachnoid Hemorrhage

**DOI:** 10.3390/medicina58020323

**Published:** 2022-02-21

**Authors:** Ieva Buce-Satoba, Daina Rozkalne, Biruta Mamaja, Gaida Krumina, Agnese Ozolina

**Affiliations:** 1 Department of Doctoral Studies, Riga Stradins University, 16 Dzirciema Str., 1007 Riga, Latvia; 2 Riga East University Hospital, 2 Hipokrata Str., 1079 Riga, Latvia; daina.rozkalne@aslimnica.lv (D.R.); gaida.krumina@apollo.lv (G.K.); agnese.ozolina@icloud.com (A.O.); 3 Department of Anesthesiology and Intensive Care, Riga Stradins University, 13 Pilsonu Str., 1002 Riga, Latvia; biruta.mamaja@aslimnica.lv; 4 Department of Radiology, Riga Stradins University, 2 Hipokrata Str., 1079 Riga, Latvia

**Keywords:** aneurysmal subarachnoid hemorrhage, secondary cerebral vasospasm, delayed cerebral ischemia, neuroinflammation, leukocytosis, C-reactive protein

## Abstract

*Background and Objectives*: Secondary cerebral vasospasm (CV) with subsequent delayed cerebral ischemia (DCI) after aneurysmal subarachnoid hemorrhage (aSAH) remains an unpredictable pathology. The aim of this retrospective study was to investigate the association between inflammatory parameters, white blood cell (WBC) count, and C-reactive protein plasma levels (CRP) and the occurrence of secondary CV in patients with aSAH. *Materials and Methods*: The medical records of 201 Intensive Care Unit patients in Riga East University Hospital with aSAH were retrospectively reviewed in a 24-month period. WBC count and CRP values were observed at admission to the hospital and on the third day. According to the inclusion criteria, 117 (48 males) participants were enrolled for further analysis, with average age of 56 ± 15 years (mean ± SD). In total, secondary CV was diagnosed in 21.4% of cases, and DCI in 22.4% of cases. The patients were classified into three groups: SAH-CV group (*n* = 25), SAH-DCI group (*n* = 12), and SAH or control group (*n* = 80), for comparative analysis. *Results*: We found that SAH-CV patients demonstrated notably higher inflammatory parameters compared to controls: WBC 13.2 ± 3.3 × 10^9^/L vs. 11.2 ± 3.7 × 10^9^/L; *p* = 0.01 and CRP median 9.3 mg/L vs. 1.9 mg/L; *p* < 0.001, respectively. We found that the odds of developing CV increased by 5% for each CRP increase of 1 mg/L at admission (OR, 1.05; CI, 1.014–1.087; *p* = 0.006). Concomitantly, the odds increased by 16% for every rise in WBC count of 1 × 10^9^/L (OR, 1.16; CI, 1.02–1.32; *p* = 0.02). WBC count was associated with the occurrence of CV with 96% sensitivity and 40% specificity, with a cut off level of 10.015 × 10^9^/L and AUC 0.683; *p* = 0.006. CRP displayed 54% sensitivity and 90% specificity with a cut off value of 8.9 mg/L and AUC 0.751; *p* < 0.001. Moreover, higher values of inflammatory parameters at admission correlated with a longer stay in ICU (r = 0.3, *p* = 0.002 for WBC count and r = 0.305, *p* = 0.002 for CRP values), and poor outcome (death) was significantly associated with higher CRP values at admission and on the third day (16.1. vs. 2.2. and 57.4. vs. 11.1, *p* < 0.001, respectively). Higher mortality was detected in SAH-CV patients (32%) compared to controls (6.3%; *p* < 0.001). *Conclusions*: Inflammatory parameters such as WBC count and CRP values at admission might be helpful to predict the development of secondary CV.

## 1. Introduction

Aneurysmal subarachnoid hemorrhage (aSAH) accounts for about 3% of all strokes [1]. The risk of disability notably increases if the patient develops secondary cerebral vasospasm (CV), which can lead to delayed cerebral ischemia (DCI), after surviving the initial bleeding [2]. Consequently, the identification of patients at risk should be prioritized with the aim of diagnosing secondary CV earlier, as previously proposed [3,4,5,6,7].

The incidence of aSAH varies by geographic region from 4 to up to 28 per 100,000 person-years [2]. In Latvia, the incidence has increased from 9 to 11 per 100,000 population within the last 5 years, according to the Centre of Diseases Prevention and Control of Latvia [8].

Neuroinflammation plays a significant role in the pathogenesis of secondary CV and DCI [9,10]. Systemic inflammatory response syndrome with leukocytosis and increased values of C-reactive protein (CRP), tumor necrosis factor alfa, interleukin-6, and others are observed in up to 60% of patients with aSAH [9,10,11]. Recently, many researchers have tried to discover the most precise clinical and laboratorial predictors and risk factors for CV and DCI [3,4,5,6,7]. A population-based study of 8346 patients found that younger age, females, smoking, compromised hemodynamics, and clinical severity were associated with the occurrence of CV [4]. Moreover, the last published large meta-analysis of 3268 patients demonstrating an association of CRP and white blood cell (WBC) count with the occurrence of DCI after aSAH showed no significant associations between an early increase in WBC count and DCI (pooled OR 1.13, 95% CI 0.95–1.34; *p* = 0.179). In contrast, this meta-analysis revealed that an early increase in CRP was significantly associated with a higher risk of occurrence of DCI (pooled OR 1.30, 95% CI 1.10–1.54; *p* = 0.002). Nevertheless, it is still unclear whether early phase CRP and WBC count may serve as prognostic markers for aSAH, and need further investigation [7].

Several studies have focused on genetical (apoprotein E, haptoglobin) and cell damage markers, altered metabolism, and vascular tone markers and microparticles—cytokines, E-selectin, tumor necrosis factor alpha—which might be helpful to stratify the risk and detect CV earlier, but none has been approved yet [4,7,9,10,11,12,13,14,15,16,17,18,19,20,21,22,23,24]. 

We hypothesized that standard inflammatory parameters—white blood cell count and CRP plasma levels—at admission and on the third day might strengthen the possibility of early prediction of CV. Consequently, by performing retrospective analysis, our primary endpoint was to address the association between WBC count and CRP values and the development of secondary CV in patients with aSAH. 

## 2. Materials and Methods

### 2.1. Study Subjects

The medical records of 201 patients with aSAH admitted to the Intensive Care Unit (ICU) of Riga East University Hospital were considered for retrospective analysis within the time period of 24 months. 

The inclusion criteria were age > 18 years; level of consciousness according to the Glasgow coma scale (GCS) of 15-5 points; confirmed cerebral aneurysm rupture on computer tomography angiography (CTA) or digital subtraction angiography (DSA); any size of SAH according to radiological classification by Fisher score I-IV, except those with intracerebral hematoma; and WBC count and CRP plasma levels measured at admission and on the third day. 

The exclusion criteria were SAH for another reason (spontaneous SAH without confirmed vascular pathology, rupture of malformation, rupture of dural arteriovenous fistula, traumatic SAH); level of consciousness by GCS < 5 points; aSAH with intracerebral hematoma (Fisher score IV with ICH); and any underlying condition affecting WBC and CRP values, such as suspected or known infection, autoimmune disease and steroid intake, malignancies including hematological diseases, and acute or recent trauma. 

The aSAH diagnosis was confirmed by a neurosurgeon after computer tomography angiography and was approved by a radiologist. Information relating to comorbidities was precisely detected from previous medical statements of the patient and from senior doctors’ reports after primary examination of the patient at admission and ICU, where the majority were able to give information about their health status and harmful habits.

**Figure 1:**
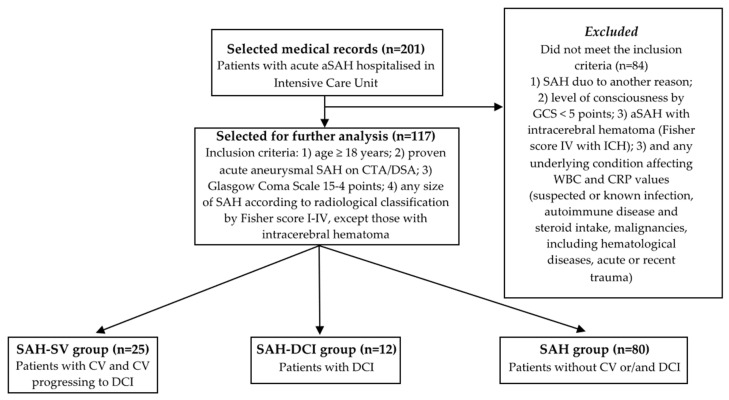
Patient selection flow chart. aSAH—aneurysmal subarachnoid hemorrhage; CTA—computer tomography angiography; DSA—digital subtraction angiography; GCS—Glasgow Coma Scale; CV—cerebral vasospasm; DCI—delayed cerebral ischemia.

Finally, 117 patients who met the inclusion criteria were enrolled for further analysis. The cohort was divided into three groups based on the occurrence of CV and DCI: the SAH-CV group (*n* = 25), which included patients with CV and CV progression to DCI as a consequence of undiagnosed/untreated/resistant to treatment CV; the SAH-DCI group (*n* = 12), including patients with confirmed DCI; and the SAH group—control group (*n* = 80), including patients without CV or DCI. Figure 1 presents a flow chart of the patient selection and classification into the three groups. 

Demographic data were manually obtained from medical records: sex, age, body mass index (BMI), comorbidities (arterial hypertension, diabetes mellitus, general atherosclerosis), regular smoking, chronic alcohol intake, as well as stay in ICU and total stay in hospital.

### 2.2. Laboratory Assays

The following laboratory parameters, WBC count (normal range 4.00–9.00 × 10^9^/L), and CRP plasma levels (normal range 0–5 mg/L) were retrospectively analyzed at two time points—at admission to the hospital and on the third day. The WBC count from whole blood in a complete blood count tube with potassium ethylenediaminetetraacetic acid was determined by Complete Blood Count with 5-Part Differential test, Automated Hematology Analyzer XN-1500, Sysmex Corporation (HQ: Kobe Japan; Chairman and CEO: Hisashi Ietsugu). The CRP value in a serology and chemistry testing tube was detected by C-reactive protein test, analyzer Cobas® 6000, Roche Diagnostics (Basel, Switzerland). 

### 2.3. Statistical Analysis

The data were analyzed with SPSS (IBM Statistics SPSS 27 version, Chicago, IL, USA). Continuous variables were presented as mean ± standard deviation (SD) and categorical variables as percentages (%). Comparisons between the groups were performed with a Mann–Whitney U test for non-parametric variables and a two-sample t test or ANOVA for parametric variables. The Chi-square test was used to analyze categorical data. We performed risk calculation with odds ratio to demonstrate the prognostic efficiency of the WBC count and CRP values for the development of CV and DCI. We performed receiver operating characteristics (ROC) curve analysis of the WBC count and CRP values, including calculations of the area under the curve (AUC) for the development of CV. Statistical significance was defined as a *p* < 0.05. 

## 3. Results

### 3.1. Patient Characteristics

Retrospectively, 117 cases of 69 (59%) females and 48 (41%) males with an average age of 56 ± 15 years (mean ± SD) who were admitted to the ICU and met the inclusion criteria were subjected to further analysis. The descriptive statistics of the cohort are presented in Table 1.

The patients were classified into three groups according to the development of CV and DCI. In total, CV was diagnosed in 21.4% of cases, but DCI was found in 22.4% of cases. Only half of the patients (*n* = 14, 54%) with DCI had a previously confirmed diagnosis of CV. More younger, male patients were detected in the SAH-DCI group compared to SAH-CV and controls. There were no differences in body mass index, as well as in detected comorbidities and harmful habits between groups. We found that patients who later experienced CV presented lower values of GCS at admission compared to controls (13 vs. 15, *p* = 0.025). Similarly, aSAH severity, according to the Fisher score, was associated with the later occurrence of CV, as depicted in Table 1.

Additionally, patients with CV spent a longer time in ICU compared to controls (8 vs. 4 days, *p* = 0.002). Consequently, mortality was the highest in the SAH-CV group (32%). 

### 3.2. Difference in Inflammatory Parameters between Three Groups

As shown in Table 2, both the WBC count values at admission (*p* = 0.01 and *p* = 0.016) and CRP values at admission (*p* < 0.001 and *p* = 0.017) were higher in the SAH-CV group compared to the SAD-DCI and SAH groups. Unexpectedly, intergroup differences could not be detected in the mean WBC count and CRP values on the third day. 

### 3.3. Role of Dynamic Changes in Inflammatory Parameters

**Figure 2:**
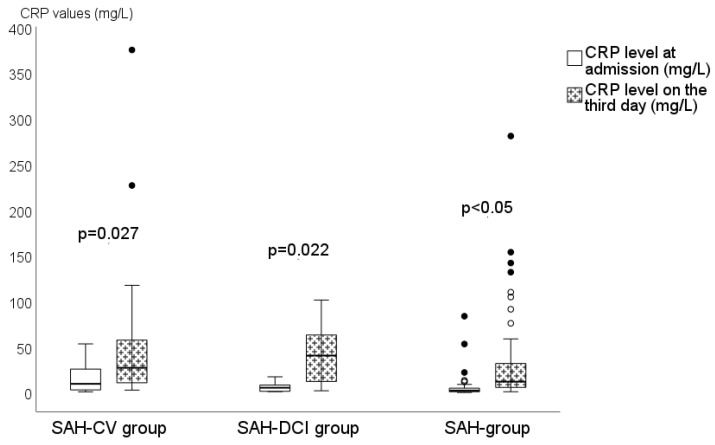
Rise of CRP between all three groups. Plasma levels of C-reactive protein at admission and on the third day in patients of SAH-CV group (patients with CV and CV progression to DCI as a consequence of undiagnosed/untreated/resistant to treatment CV), SAH-DCI (patients with confirmed DCI without previously diagnosed CV), and SAH group or controls (patients without CV and DCI). CRP—C-reactive protein; mg—milligram; L—liter. Data presented as median. Black and white dots—outliers in the data.

In all three groups, we observed a statistically significant rise of CRP values from admission to the third day (*p* = 0.027 in the SAH-CV group, *p* = 0.022 in the SAH-DCI group, and *p* < 0.001 in the SAH group), as seen in Figure 2. However, an increase in the mean WBC count between the two analyzed time points was not detected.

In total, we found that a CRP increase for each 1 mg/L at admission increases the odds to develop CV by 5% (OR, 1.05; CI, 1.014–1.087; *p* = 0.006). Concomitantly, we found that a WBC count increase for each 1 × 10^9^/L at admission increases the odds to develop CV by 16% (OR, 1.16; CI, 1.02–1.32; *p* = 0.02).

**Figure 3:**
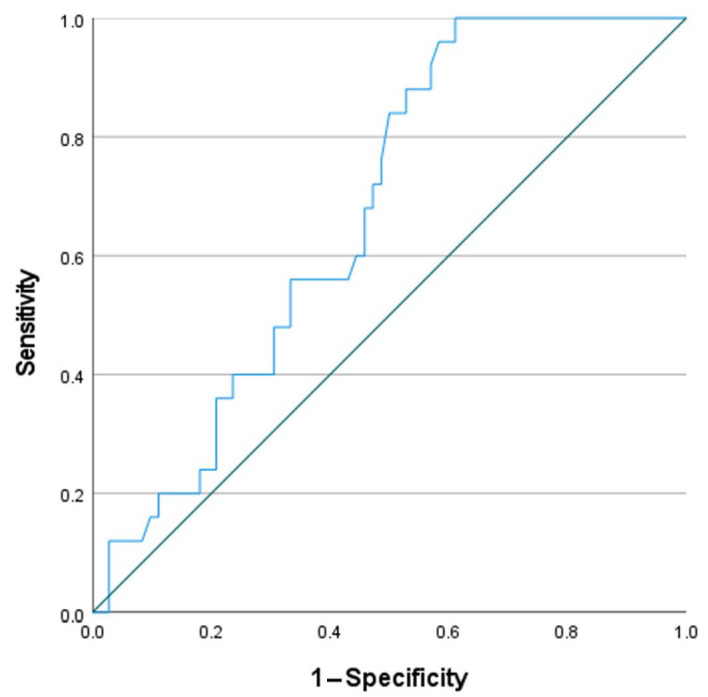
ROC curve showing sensitivity and specificity of WBC count at admission and occurrence of cerebral vasospasm (*p* = 0.006).

**Figure 4:**
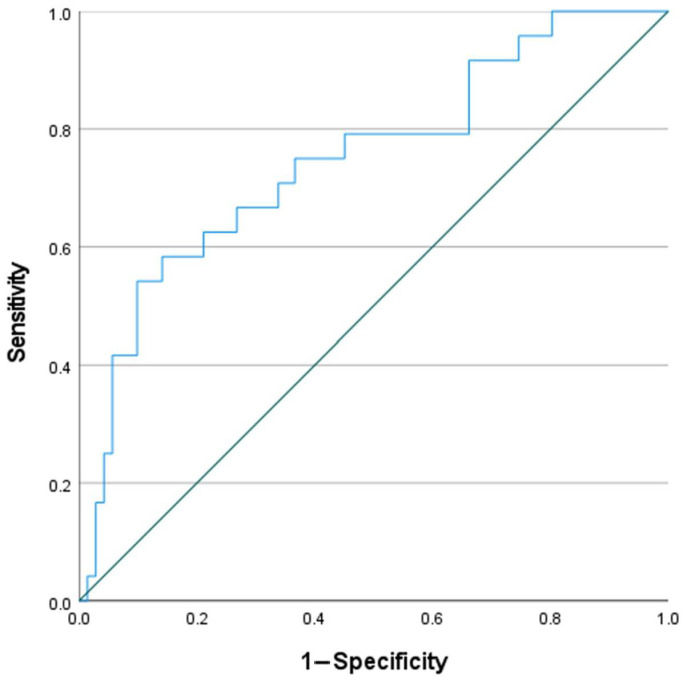
ROC curve showing sensitivity and specificity of CRP values at admission and occurrence of cerebral vasospasm (*p* < 0.001).

In aSAH-CV patients, a ROC analysis showed 96% sensitivity and 40% specificity for an association with WBC count at admission with a cut off value of 10.015 × 10^9^/L (AUC 0.683; *p* = 0.006), as depicted in Figure 3. A more significant association was found with CRP values determined at the same time point, with a sensitivity and specificity of 54% and 90%, respectively, with a cut of value of 8.95 mg/L (AUC 0.751; *p* < 0.001), as shown in Figure 4. 

Additionally, we discovered a positive correlation between higher levels of analyzed inflammatory parameters at admission and days stayed in ICU (r = 0.3, *p* = 0.002 for WBC count and r = 0.305, *p* = 0.002 for CRP values).

When analyzing those with poor outcome (death), we found that the CRP values at admission and on the third day were significantly higher (16.1. vs. 2.2. and 57.4. vs. 11.1, respectively; *p* < 0.001). Higher CRP values for every 1 mg/L at admission increases the odds of a poor outcome by 5.5% (OR, 1.055; CI, 1.010–1.103; *p* = 0.017). An association between WBC count and poor outcome was not found. 

## 4. Discussion

Although the underlying mechanism of secondary CV still is not fully clarified, there is growing evidence that neuroinflammation plays a significant role [9,10,11,25]. During aSAH, peripheral immune cells are recruited and activated with a parallel production of neutrophils [26,27]. Therefore, WBC count and CRP plasma levels were extracted for our retrospective analysis as potential predictors of early CV.

In this study, we discovered that there is a relationship between WBC count and CRP values at admission and the development of secondary CV. Therefore, we speculate that WBC count and CRP plasma levels at admission may be used as additional prognostic factors to identify patients at a higher risk of the occurrence of CV. Our finding is confirmed by the significantly higher values of WBC count and CRP at admission for patients who later experienced secondary CV, and by the odds of developing secondary CV when the CRP value increases (5% for every 1 mg/L) and the WBC count increases (16% for every 1 × 10^9^/L). Additionally, the ROC analysis showed that WBC count at admission has very high sensitivity (96%), but low specificity (40%); conversely, the CRP values at the same time point demonstrated a low sensitivity (54%), but higher specificity (90%) for the prediction of CV. 

Researchers have focused on different biomarkers and associations with the development of CV and DCI [4,7,13,14,15,16,17,18,19,20,28], but so far without significant preferences. Authors have tried to confirm the association between elevated CRP and DCI in a recent large meta-analysis of 3268 patients, but also concluded that whether early phase CRP and WBC count may serve as prognostic markers for aSAH needs more investigation [7]. 

A prospective study conducted by Hurt et al. demonstrated that serum C-reactive protein levels were higher in patients with severe vasospasm during the period of vasospasm, confirming that occurrence of CV occurs in parallel with inflammatory processes [21]. Another study, by Fountas et al., showed higher CRP values from the day of admission to the ninth day in patients with CV or poor outcomes [21]. In our study, the CRP levels at admission were higher and continued to rise from a median of 9.3 mg/L at admission up to 26.3 mg/L on the third day for patients in whom secondary CV was detected. Moreover, CRP presented high specificity (90%) for the prediction of CV; however, CRP values can only be a diagnostic tool to detect aSAH and CV in half the cases.

Secondary CV usually occurs from the third day after aSAH and peaks at day six and eight [29]. In addition, according to other studies, CRP peaks on the third day [22,23]. We focused our attention on how to predict the occurrence of secondary CV as early as possible. Therefore, time points at admission and on the third day of WBC count and CRP determination were selected based on the fact that acute phase proteins, including CRP, are released from hepatocytes in the early days after the event stimulated by IL-6 as a response to neutrophil production [27].

We retrospectively observed that CV was diagnosed in 21.4% of all cases, but DCI in 22.4%. CV can be detected in angiography up to 70%, but only around 20–30% develop clinically significant CV that could lead to DCI [30]. Incidence in other studies fluctuates from 13.6 to 36.6% for CV and 15.2 to 41.5% for DCI [17,18,19,21,22]. Thus, we conclude that our data are consistent with those obtained in other studies.

According to recent studies, several scores like the Hunt–Hess score, GCS, the World Federation of Neurological Surgeons Scale, and the Fisher scale can also predict the outcome of aSAH patients [31,32]. Unfortunately, none of the previously mentioned grading systems have high sensitivity or specificity. The clinical classification grades of aSAH only predict outcome [32,33], but radiological classification scores like the Fisher score may only be useful in predicting the occurrence of CV [34]. There is no score that is used to predict DCI [31]. 

We must admit that we found a statistically significant rise of CRP values on the third day in all three analyzed groups (*p* = 0.027 in the SAH-CV group, *p* = 0.022 in the SAH-DCI group, and *p* < 0.001 in the SAH group). We believe that this finding may be influenced by a variety of other factors like aspiration pneumonia and coagulation activation. Aspiration pneumonia occurs in 20–60% of critically ill patients and CRP increases more rapidly within the first 48 h in patients with aspiration pneumonia [35]. This theory could be indirectly supported by the fact that an association was found between the severity of aSAH according to GCS and higher inflammatory laboratory parameters, whereas severe aSAH is an independent risk factor for aspiration. Similarly, blood coagulation is activated 72 h after aSAH onset [36]. 

Nevertheless, we aimed to retrospectively identify additional, simple laboratory markers that could help to predict higher risk for the development of CV and DCI and might be useful in identifying patients who need more precise monitoring after aSAH in ICU. Currently, different monitoring methods [37,38,39,40,41,42,43,44] are available, but none are adjusted as a standard in aSAH patients. Transcranial doppler ultrasonography is the gold standard for detecting CV in aSAH patients. It is a non-invasive, safe, easily repeatable bedside technique [37,38,39]. At the same time, it has some limitations, such as technical and logistic pitfalls, and it is operator-dependent. Furthermore, not all patients have a good acoustic window [39,40]. Continuous electroencephalography may also be used for monitoring aSAH patients for DCI and seizures, but not for CV [41]. Similarly, cerebral micro-dialysis monitoring may be used for the detection of cerebral ischemia [42], but it is an invasive method and is not routinely used [43]. Near-infrared spectroscopy seems promising, which is a continuous, non-invasive bedside monitoring method of regional cerebral oxygen saturation, that can help in the early detection of hypoperfusion of the brain, but the data are controversial [44]. 

We admit that this retrospective study has several limitations. The patient selection could have been influenced by unprecise data from medical histories or our interpretation. We admit that we are missing full data for approximately 6% of cases, as shown in Table 1. To limit information bias about comorbidities and harmful habits, we approved that the majority of the patients were able to give information about their health status at admission. Subsequently, the data were precisely manually collected going through each medical history combined with the data from electronic databases and from the participants. Nevertheless, out of 201 selected medical records, 117 were selected for further analysis. Unfortunately, 84 patients did not meet the inclusion criteria, as depicted in the flow chart in Figure 1. The main reasons were SAH for another cause, or that cerebral aneurysm was not found on computer tomography angiography or later during the provided digital subtraction angiography. Additionally, we excluded patients with SAH according to the Fisher score IV with intracerebral hematoma because they are more likely to have ultra-early development of CV [5]. This resulted in a smaller sample size of the SAH-DCI group. Important confounders like aspiration pneumonia, ventricular drainage insertion with subsequent ventriculitis, or discoagulation might have an effect on the WBC count and CRP values, and might affect our results. Unfortunately, we were not able to confirm or deny the effect of cofounders on the WBC count and CRP values using Binary Logistic Regression and Multinominal Logistic Regression analysis due to the small group size. 

To confirm our hypothesis, a broader retrospective or prospective study is needed in this direction. 

## 5. Conclusions

Inflammatory parameters such as WBC count and CRP values at admission may be used as additional prognostic factors, along with widely used clinical and radiological classification scores, to identify patients who have a higher risk of developing secondary CV after aSAH. Moreover, they may be used as additional prognostic factors to select patients who need more attention and monitoring in ICU. 

## Figures and Tables

**Table 1:** Descriptive statistics of patients with aSAH admitted to Intensive Care Unit.

Variables	Totally *n* = 117	SAH-CV Group *n* = 25	SAH-DCI Group *n* = 12	SAH Group (Controls) *n* = 80	*p*-Value
Demographic data					
Male sex, *n* (%)	48 (41)	13 (52)	9 (75) ^&^	26 (32.5)	0.005 ^&^
Age, yr (mean ± SD)	56 ± 15	53 ± 13	47 ± 14 ^&^	58 ± 157	0.022 ^&^
BMI, kg/m^2^ (mean ± SD) *	26 ± 4	25.5 ± 3.5	26 ± 4	26 ± 4	NS
Comorbidities					
Arterial hypertension, *n* (%) *	70 (63.6)	14 (63.6)	5 (45.5)	51 (66.2)	NS
Diabetes mellitus, *n* (%) *	6 (5.4)	1 (4.3)	0 (0)	5 (6.5)	NS
General atherosclerosis, *n* (%) *	16 (14.7)	2 (8.7)	2 (18.2)	12 (16)	NS
Harmful habits					
Smoking, *n* (%) *	14 (24.6)	2 (8.7)	4 (50)	8 (19.5)	NS
Chronic alcohol intake, *n* (%) *	7 (10.8)	2 (20)	0 (0)	5 (10.9)	NS
SAH severity according to scores					
GCS, points (median)	15	13 ^#^	15	15 ^#^	0.025 ^#^
Fisher score grade, *n* (%)					
I	3 (2.6)	0 (0)	0 (0)	3 (3.8)	NS
II	10 (8.5)	1 (4) ^#^	2 (16.7) ^&^	7 (8.8) ^#^	0.011 ^#^
					0.003 ^&^
III	30 (25.6)	7 (28) ^#,$^	5 (41.7) ^&,$^	18 (22.5) ^#^	0.003 ^#^
					<0.001 ^&,$^
IV (without ICH)	74 (63.2)	17 (68) ^#,$^	5 (41.7) ^&,$^	52 (65) ^#,&^	<0.001 ^#,&,$^
Mortality, *n* (%)	14 (12)	8 (32) ^#^	1 (8.3)	5 (6.3) ^#^	<0.001 ^#^
Length of stay in hospital, days (median)					
Days in ICU	4	8 ^#^	5	4 ^#^	0.002 ^#^
Total stay	18	23	21	17.5	NS

* Full data on all patients were not available: on average, data were not available in 6% of cases regarding comorbidities and in 23% of cases regarding harmful habits. yr—years; kg—kilogram; SAH—subarachnoid hemorrhage; CV—cerebral vasospasm; DCI—delayed cerebral ischemia; BMI—body mass index; GCS—Glasgow coma scale; ICH—intracerebral hemorrhage; ICU—intensive care unit; NS—not significant. *p*-values: ^#^ comparing SAH-CV group with SAH group; ^&^ comparing SAH-DCI group with SAH group; ^$^ comparing SAH-CV group with SAH-DCI group.

**Table 2:** WBC count and CRP values at admission and on the third day between the three groups.

Laboratory Parameters	Totally *n* = 117	SAH-CV Group *n* = 25	SAH-DCI Group *n* = 12	SAH Group (Controls) *n* = 80	*p*-Value
WBC count at admission (×10^9^/L) (mean ± SD)	11.7 ± 3.8	13.2 ± 3.3 *^,$^	11.3 ± 4.7 ^$^	11.2 ± 3.7 *	0.01 *, 0.016 ^$^
WBC count on the third day (×10^9^/L) (mean ± SD)	11.6 ± 3.6	12.4 ± 3.5	12.5 ± 3.9	11.1 ± 3.7	NS
CRP values at admission (mg/L) (median)	2.95	9.3 *^,$^	1.1 ^$^	1.9 *	<0.001 *, 0.017 ^$^
CRP values on the third day (mg/L) (median)	15.5	26.3	25.5	11.67	NS

SAH—subarachnoid hemorrhage; CV—cerebral vasospasm; DCI—delayed cerebral ischemia; WBC—white blood cell; CRP—C-reactive protein; mg—milligram; L—liter, NS—not significant. *p*-values: * comparing SAH-CV group with SAH group; ^$^ comparing SAH-DCI group with SAH group.

## Data Availability

The data presented in this study are available from the corresponding author on reasonable request.
